# Profile of Basal Cell Carcinoma Mutations and Copy Number Alterations - Focus on Gene-Associated Noncoding Variants

**DOI:** 10.3389/fonc.2021.752579

**Published:** 2021-11-25

**Authors:** Paulina Maria Nawrocka, Paulina Galka-Marciniak, Martyna Olga Urbanek-Trzeciak, Ilamathi M-Thirusenthilarasan, Natalia Szostak, Anna Philips, Laura Susok, Michael Sand, Piotr Kozlowski

**Affiliations:** ^1^ Department of Molecular Genetics, Institute of Bioorganic Chemistry, Polish Academy of Sciences, Poznan, Poland; ^2^ Laboratory of Bioinformatics, Institute of Bioorganic Chemistry, Polish Academy of Sciences, Poznan, Poland; ^3^ Department of Dermatology, Venereology and Allergology, St. Josef Hospital, Ruhr-University Bochum, Bochum, Germany; ^4^ Department of Plastic Surgery, St. Josef Hospital, Catholic Clinics of the Ruhr Peninsula, Essen, Germany Department of Plastic, Reconstructive and Aesthetic Surgery, St. Josef Hospital, Essen, Germany

**Keywords:** basal cell carcinoma (BCC), cancer somatic mutations, noncoding mutations, immune checkpoint, copy number alterations, cancer drivers, TERT, DPH3

## Abstract

Basal cell carcinoma (BCC) of the skin is the most common cancer in humans, characterized by the highest mutation rate among cancers, and is mostly driven by mutations in genes involved in the hedgehog pathway. To date, almost all BCC genetic studies have focused exclusively on protein-coding sequences; therefore, the impact of noncoding variants on the BCC genome is unrecognized. In this study, with the use of whole-exome sequencing of 27 tumor/normal pairs of BCC samples, we performed an analysis of somatic mutations in both protein-coding sequences and gene-associated noncoding regions, including 5’UTRs, 3’UTRs, and exon-adjacent intron sequences. Separately, in each region, we performed hotspot identification, mutation enrichment analysis, and cancer driver identification with OncodriveFML. Additionally, we performed a whole-genome copy number alteration analysis with GISTIC2. Of the >80,000 identified mutations, ~50% were localized in noncoding regions. The results of the analysis generally corroborated the previous findings regarding genes mutated in coding sequences, including *PTCH1*, *TP53*, and *MYCN*, but more importantly showed that mutations were also clustered in specific noncoding regions, including hotspots. Some of the genes specifically mutated in noncoding regions were identified as highly potent cancer drivers, of which *BAD* had a mutation hotspot in the 3’UTR, *DHODH* had a mutation hotspot in the Kozak sequence in the 5’UTR, and *CHCHD2* frequently showed mutations in the 5’UTR. All of these genes are functionally implicated in cancer-related processes (e.g., apoptosis, mitochondrial metabolism, and *de novo* pyrimidine synthesis) or the pathogenesis of UV radiation-induced cancers. We also found that the identified *BAD* and *CHCHD2* mutations frequently occur in melanoma but not in other cancers *via* The Cancer Genome Atlas analysis. Finally, we identified a frequent deletion of chr9q, encompassing *PTCH1*, and unreported frequent copy number gain of chr9p, encompassing the genes encoding the immune checkpoint ligands PD-L1 and PD-L2. In conclusion, this study is the first systematic analysis of coding and noncoding mutations in BCC and provides a strong basis for further analyses of the variants in BCC and cancer in general.

## Introduction

Basal cell carcinoma (BCC), a type of nonmelanoma skin cancer, is the most common human cancer affecting predominantly elderly people of the Caucasian population (1–3). The lifetime risk of BCC in the Caucasian population is ~30%, and it is higher in men and fair-skinned people. BCC usually occurs sporadically but can also develop as a result of Gorlin syndrome (also known as nevoid basal cell carcinoma syndrome), an autosomal dominant hereditary condition with an incidence of approximately 1:30,000 (4) characterized by the frequent appearance of multiple BCC lesions that develop at a younger age together with skeletal abnormalities, odontogenic keratocysts, and an increased risk of medulloblastoma. Histologically, BCCs are classified into three major subtypes: nodular, which is the most common subtype; superficial; and infiltrative or sclerodermiform. Other subtypes as well as mixed types occur less frequently (5–7). Predominantly, superficial and nodular BCCs are slow-growing, locally invasive, epidermal tumors with a metastasis rate of <0.1% (8, 9), while infiltrative BCCs are characterized by more aggressive, tong-like, subclinical growth patterns mimicking icebergs, as they often grow below clinically healthy-looking skin (10, 11). Although BCC aggressiveness and metastatic potential are overall low, the commonness of BCC and the increasing incidence associated predominantly with aging populations has brought attention to its pathogenesis (2, 3, 12–17). Exposure to ultraviolet (UV) radiation, which can lead to point mutations frequently represented by C>T and CC>TT transitions, is the main causative factor in the pathogenesis of BCC (18). Additional risk factors include ionizing radiation, arsenic ingestion, and immune suppression (19, 20).

BCC is characterized by the highest mutation rate observed among cancers, having over 65 mutations/Mbp (14, 15). The most frequent genetic alterations occurring in BCC are mutations disturbing the hedgehog (SHH/PTCH1/SMO) pathway, predominantly loss-of-function mutations in *PTCH1* but also activating mutations in *SMO*; these genes encode two transmembrane proteins, PTCH1 (also known as Patched1) and SMO (also known as Smoothened), respectively (14, 15). The pathway is activated by the SHH signaling protein (also known as Sonic hedgehog), which binds to the extracellular domain of PTCH1, disabling inhibition of SMO; this in turn activates GLI transcription factors. Germline mutations in *PTCH1* predispose patients to Gorlin syndrome (21).

Previous studies, including whole-exome sequencing (WES) analyses, have also recognized other genes/pathways frequently mutated in BCC, including *TP53, MYCN, PPP6C, PTPN14, STK19*, and *LATS1* (14, 15), as well as genes involved in the RTK-RAS-PI3K and Hippo-YAP pathways (15). However, as an overwhelming majority of BCC genetic studies (as well as those in other cancers) have focused almost exclusively on protein-coding sequences, very little is known about mutations in noncoding regions (noncoding mutations). Noncoding mutations are not studied/reported even if detected, e.g., as a result of WES. On the other hand, it is well known that the noncoding parts of genes, i.e., promoters, introns, or 5’ and 3’ untranslated regions (5’UTRs and 3’UTRs, respectively), encompass numerous functional elements important for the proper functioning of the genes (22–24). Somatic mutations may disrupt or modify the properties of these elements, acting either as gain- or loss-of-function mutations and thus enhancing/accelerating or switching off the function of some genes. Despite the limited number of studies on noncoding mutations, there are some spectacular examples of noncoding driver mutations, for example, *TERT* promoter mutations, which occur most frequently in melanoma, brain, and bladder cancers but are also reported in BCC (25–27), and mutations in the precursor of miR-142, which frequently occur in non-Hodgkin lymphomas and acute myeloid leukemia [summarized in (28)]. The miRNA biogenesis enzyme DICER has also been shown to bear mutations that could play a role in aberrant miRNA expression in BCC (29–31). It should also be noted that an effort to catalog cancer somatic mutations in the noncoding genome has recently been undertaken (32, 33); however, this pancancer project does not include BCC.

To preliminarily explore the occurrence of noncoding somatic mutations in BCC, we performed WES of over two dozen BCC samples, extending the analysis beyond protein-coding sequences and focusing on gene-associated noncoding regions, i.e., 5’UTRs, 3’UTRs, and exon-adjusted sequences of introns, covered by standard WES approaches. Apart from the fact that our results well-replicate those of previous BCC studies in terms of mutations in protein-coding genes, we showed that a substantial portion of mutations is located in noncoding regions. Many of these mutations frequently recur in particular noncoding regions or in specific hotspot positions. Computational analyses showed that some of the gene mutations in noncoding regions are potential cancer drivers and are functionally related to skin cancers. Additionally, whole-genome copy number alteration (CNA) analysis revealed frequent deletion of chr9q, encompassing *PTCH1*, and unreported frequent amplification of chr9p, including the genes encoding two immune checkpoint ligands PD-L1 and PD-L2.

## Materials and Methods

### Sample Collection and DNA Preparation

A total of 27 pairs of tissue (tumor and normal adjacent healthy skin) were collected from the Department of Plastic Surgery, St. Josef Hospital, Catholic Clinics of the Ruhr Peninsula, Essen, Germany. While excising the BCC tissues with cold steel under local anesthesia, 4-mm punch biopsies were taken from the center of the tumor and from nonlesional epithelial skin (as normal, intraindividual controls). These samples were immediately placed in RNAlater (Qiagen, Hilden, Germany) and stored at −80°C. Tissue homogenization was performed with stainless steel beads of 5 mm (Qiagen) and TissueLyser LT (Qiagen). DNA was extracted with an AllPrep DNA/RNA/miRNA Universal Kit (Qiagen) according to the manufacturer’s protocol. All samples were quantified using a NanoDrop One (Thermo Scientific, Waltham, USA) and Qubit fluorometer 3.0 (Invitrogen) (Qubit dsDNA HS Assay (Life Technologies, Carlsbad, USA)), and DNA size and quality were tested using gel electrophoresis.

### Exome Sequencing and Data Processing

The library was prepared with 200 ng of high-quality DNA using the SureSelectXT Library Prep Kit (Agilent). A SureSelectXT Human All Exon V6 kit (Agilent) was used for exome capture. Sequencing was performed on an Illumina NovaSeq 6000 (San Diego, USA), generating 2x 100 bp paired-end reads. Library preparation, exome enrichment, and sequencing were performed at CeGaT, Tuebingen, Germany. Demultiplexing of the sequencing reads was performed with Illumina bcl2fastq (2.19). Adapters were trimmed with Skewer (version 0.2.2) (34). The Phred score was given with Illumina standard Phred encoding (offset +33). For each sample, two FASTQ files corresponding to forward and reverse reads were obtained. Next steps were done by us on the Poznan Supercomputing and Networking Center (PSNC) Eagle supercomputer. Paired-end reads were aligned to hg38 using BWA. PCR duplicates were marked and removed with the Picard package. Indel realignments with known sites and base quality score recalibration were performed with GATK version 4.1.2.0. SAM to BAM conversion was done using SAMtools. Somatic single-nucleotide variants were called with MuTect2 (version 4.1.0.0. with the use of the tumor-normal mode). Additionally, to avoid false-positive somatic mutations, we performed filtering for germline variants present in the gnomAD database (version 2.1.1). We also generated and flagged variants with a panel of normals (PoN) comprising variants representing commonly occurring sequencing noise that may mimic low allele-fraction somatic variants. We also added information about the localization of mutations in gene subregions (CDS, 5’UTR, 3’UTR, or introns) by use of an in-house Python script. From the list of somatic mutations, we additionally removed those that did not fulfill the following criteria: (i) at least five alternative allele-supporting reads in a tumor sample; (ii) frequency of alternative allele-supporting reads in a tumor sample of at least 0.05; and (iii) frequency of alternative allele-supporting reads in the tumor sample at least 5× higher than that in the corresponding normal sample.

#### Validation of Mutations and Sequencing of the *TERT* and *DPH3* Promoters

A panel of 51 mutations detected by WES was validated by Sanger sequencing of the appropriate PCR fragments amplified with primers shown in **Table S1**. The primers used for amplification and sequencing of the *TERT* and *DPH3* promoters are shown in **Table S1**. All fragments were sequenced in two directions with the BigDye v3.1 kit (Applied Biosystems, Foster City, CA, USA), and the sequencing reactions were separated with capillary electrophoresis (POP7 polymer; ABI Prism 3130xl apparatus; Applied Biosystems, Foster City, CA, USA) according to the standard manufacturer’s recommendations.

### Mutational Signature Analysis

To analyze mutational signatures, we used the web application Mutational Signatures in Cancer [MuSiCa; http://bioinfo.ciberehd.org/GPtoCRC/en/tools.html (35)], allowing the visualization of the somatic mutational profile of each analyzed sample and estimation of the contribution values of the predefined mutational signatures [(36); Catalogue Of Somatic Mutations In Cancer, COSMIC 2020]. Samples BCC14 and BCC21 were excluded from the signature analysis due to an insufficient number of mutations.

### Identification of Hotspots, Frequently Mutated Genes, and Cancer Drivers

We defined genomic positions mutated in at least 3 (>10%) samples as hotspots. Mutations occurring in directly adjacent nucleotides were merged into one hotspot.

We defined genes with nonsynonymous mutations in a coding region in at least 5 samples, with mutations in a 5’UTR, in at least 4 samples, with mutations in a 3’UTR in at least 4 samples, and with mutations in introns (up to 40 nt from exon/intron boundaries) in at least 5 samples as frequently mutated. From the analysis, we excluded genes known to be commonly hypermutated with passenger mutations as a result of the increased background mutation rate but not related to cancer, listed in (37). To distinguish synonymous from nonsynonymous mutations, we used the SnpEff - genetic variant annotation and functional effect prediction toolbox (38), available on the Subio platform (Subio, Inc., Kagoshima, Japan, http://www.subio.jp). We also considered splice-site mutations located in introns up to +/-2 nt from exons as coding region mutations.

OncodriveFML (39) was run using the CADD score (hg38, version 1.6). The signature method was set as a complement, the statistical method was set to “amean”, and indels were included in the analysis using a max method (max_consecutive was set to 7 as default).

### Copy Number Analysis

To identify chromosome arm-level and focal regions that were significantly amplified or deleted, we used GISTIC2 (40) with the following parameters: threshold for copy number amplifications and deletions, 0.2; confidence level to calculate the region containing a driver, 0.9; broad-level analysis; and the arm peel method to reduce noise.

To validate CNAs involving chromosome 9, i.e., chr9p duplications/amplifications (affecting *JAK2*, *PDL1*/*CD274*, and *PDL2*/*CD273*) and chr9q deletions (affecting *PTCH1*), we designed and generated an MLPA assay covering the entire chromosome 9. In total, the assay consisted of 20 probes, including (i) 7 probes distributed over the chr9p (n=5) and chr9q (n=2) arms, 2 probes located in or in close proximity to *JAK2*, *PDL2*, *PDL1*, and *PTCH1* (in total 8 gene-specific probes), and 5 control probes (located on different chromosomes outside of chromosome 9 and regions of known cancer-related genes). The sequences and detailed characteristics of all probes as well as their exact positions are shown in **Table S2**.

The MLPA probes and the probe-set layout were designed according to a previously proposed and well-validated strategy (41, 42). Shortly, each probe was composed of two half-probes of equal size, and the total probe length ranged from 93 to 172 nt. The target sequences for the probes were selected to avoid common SNPs, repeat elements, and sequences of extremely high or low GC content. The MLPA probes were synthesized by IDT (Skokie, IL, USA). The MLPA reactions were run according to the manufacturer’s general recommendations (MRC-Holland, Amsterdam, the Netherlands). All reagents except the probe mixes were purchased from MRC-Holland (http://www.mlpa.com). The products of the MLPA reaction were subsequently diluted 20x in HiDi formamide containing GS Liz600, which was used as a DNA sizing standard, and separated *via* capillary electrophoresis (POP7 polymer) in an ABI Prism 3130XL apparatus (Applied Biosystems, Carlsbad, CA, USA). The obtained electropherograms were analyzed using GeneMarker software v2.4.0 (SoftGenetics, State College, PA, USA). For each individual sample, the signal intensity of each probe was divided by the geometric average signal intensity of the control probes to normalize the run-to-run signal variation, and then the normalized signal of each probe in cancer samples was divided by the corresponding signal in the corresponding normal samples and multiplied by 2. The final MLPA result of each sample is presented on a bar-plot, in which the bars show the relative copy number value of the subsequent probes.

### TCGA Analysis

To compare the mutations recurring in BCC with mutations in other cancers, we used WES-generated somatic mutation datasets of 10,369 samples representing 33 cancer types generated and deposited in the TCGA repository (http://cancergenome.nih.gov). The full names and abbreviations of all TCGA cancer types are shown in **Table S3**. Somatic mutations were identified against matched normal samples with the use of the standard TCGA pipeline (including the Mutect2, Muse, Varscan, and SomaticSnipper algorithms). We extracted somatic mutation calls (with PASS annotation only) localized in the annotated exons of *BAD*, *DHODH*, *CHCHD2*, *FLG*, and *FLG2* (exon sequences were extended by 2 nt to enable identification of intronic splice-site mutations). The extraction was performed as described in our earlier study (43) with a set of in-house Python scripts available at (https://github.com/martynaut/mirnaome_somatic_mutations).

### Mutations Visualization

All mutations were annotated according to HGVS nomenclature (at the transcript and protein levels), and the effects of mutations were defined using the Ensembl Variant Effect Predictor (VEP) tool. For visualization of mutations on gene maps, we used ProteinPaint from St. Jude Children’s Research Hospital – PeCan Data Portal (44). The protein domains visualized on gene maps were positioned according to UniProt data (45). The comutation plot showing frequently mutated genes was created with the use of the Python library CoMut (46).

### Analysis of RNA Regulatory Motifs

Target predictions were performed with the TargetScan Custom (release 5.2) web tool (47). The secondary RNA structures were predicted using mfold software (48) with default parameters. RNA sequence/structure functional motifs and transcription factor binding sites were analyzed with the RegRNA 2.0 (49) and MotifMap (50) web tools.

### Statistics

Specific statistical tests are indicated in the text, and a *p*-value <0.05 was considered significant. If necessary, *p*-values were corrected for multiple tests with the Benjamini-Hochberg procedure.

## Results

### Overall Sequencing and Mutation Occurrence Characterization

**Figure 1:**
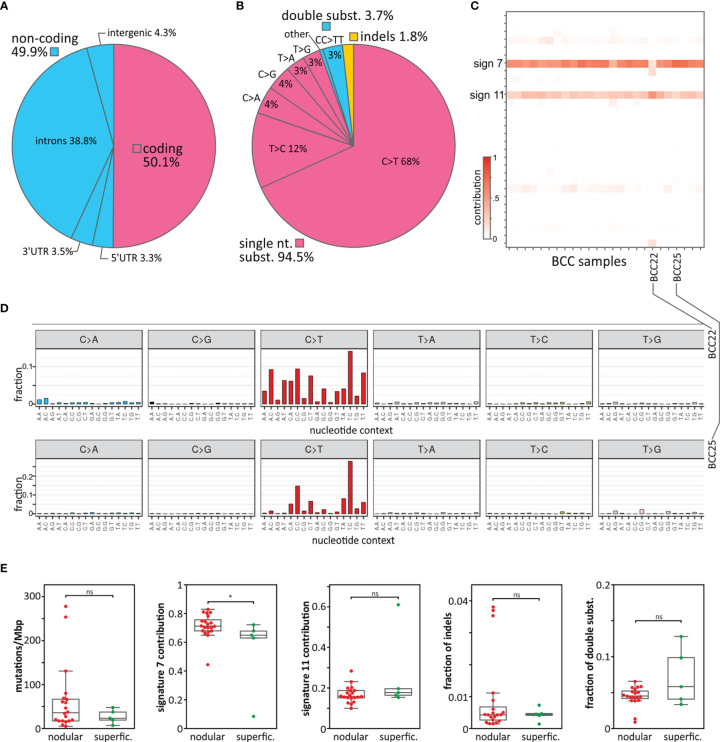
Mutation distribution, mutational signatures, and comparison of superficial and nodular BCC subtypes. **(A)** Frequency of mutations in particular gene/genomic regions. **(B)** Frequency of mutation types. **(C)** Heatmap showing the contribution of the mutational signatures (rows) to the analyzed BCC samples (columns). Higher color intensity indicates a higher contribution (as indicated on the scale bar). **(D)** Representative mutation distribution plots of samples with a high association with signature 7 (sample BCC25) and signature 11 (sample BCC22). **(E)** Comparison of nodular and superficial BCC samples in terms of (from the left) mutational load, signature 7 and signature 11 contributions, frequency of indels, and frequency of double substitutions. *P < 0.05; ‘ns’ represents that the difference is not statistically significant.

We performed WES on 27 paired tumor and corresponding intraindividual control skin DNA samples isolated from 22 nodular and 5 superficial BCC subtypes and corresponding healthy skin tissue. The average coverage of the targeted regions was 183x (185x in normal and 180x in tumor samples), ranging in different samples from 134x to 232x. In total, we identified 84,571 cancer-sample-specific somatic mutations (**Table S4**), of which 42,380 (50.1%) were located in protein-coding (coding) regions, and the remaining 42,191 (49.9%) were located in noncoding regions (**Table 1** and **Figure 1A**). The noncoding regions included (i) 5’UTRs, (ii) ~100 bp fragments of 3’UTRs adjacent to coding sequences (3’UTRs), (iii) exon-adjacent ~100 bp fragments of introns (introns), and (iv) sequences other than those classified above (i-iii), mostly intergenic sequences located upstream and downstream of the first and last gene exons (intergenic regions) (51). The average coverage of the mutated positions was 169x and was slightly higher in coding (195x) than in noncoding regions (142x), whereas the average fraction of reads mapping to alternative alleles was 0.35 (0.33 in coding and 0.40 in noncoding regions). The average mutation rate calculated based on the coding regions was 52.8 mutations/Mbp (ranging from 0.1 to 287.5), which, although slightly lower than that observed before in BCC (15, 52), is still higher than that in any other tested cancer type. Although somewhat counterintuitive, the lower mutation burden in our study than in other BCC studies (15, 52) may result from the much higher sequencing coverage in our study, which gave us much higher statistical power to filter out the fraction of false-positive mutations. The lower mutation burden in our study may also be explained by the identification in our cohort of two samples with an extremely low mutational burden (<0.2 mutations/Mbp). Most of the identified mutations were single-nucleotide substitutions (79,960 (94.5%), predominantly C>T transitions), followed by double substitutions (3,128 (3.7%), predominantly CC>TT transitions) and short (<4 nt) indels (1.483 (1.8%)) (**Table 1** and **Figure 1B**). The higher frequency of indels in noncoding regions most likely results from the excess of low complexity sequences, which cause polymerase slippage.

**Table 1:** Summary of somatic mutation distribution and mutation types in BCC.

Genomic regions	No. (%) of mutations	Average coverage of mutation positions	Average alternative allele fraction	No. (%) of substitutions	No. (%) of double substitutions	No. (%) of short indels
all mutations	84,571 (100)	169	0.35	79,960 (94.5)	3128 (3.7)	1483 (1.8)
coding	42,380 (50.1)	195	0.33	40,503 (95.6)	1697 (4.0)	180 (0.4)
noncoding	42,191 (49.9)	142	0.37	39,457 (93.5)	1431 (3.4)	1303 (3.1)
introns	32,805 (38.8)	136	0.38	30,655 (93.4)	1076 (3.3)	1074 (3.3)
3’UTR	2,926 (3.5)	154	0.39	2,721 (93.0)	100 (3.4)	105 (3.6)
5’UTR	2,832 (3.3)	163	0.37	2,650 (93.6)	125 (4.4)	57 (2.0)
intergenic region	3,628 (4.3)	167	0.35	3,431 (94.6)	130 (3.6)	67 (1.8)

To estimate the fraction of false-positive mutations, we resequenced (with Sanger sequencing) 52 mutations representing different types of alterations, including 39 substitutions and 13 indels (**Table S5**). The analysis confirmed 51/52 of the mutations, indicating a very low (2%) fraction of false-positive mutations. The fraction may be even lower, as the only unconfirmed mutation (double substitution CC>TT in *MYCN*) was present in a low fraction of reads (7%), which is generally beyond the sensitivity of Sanger sequencing.

### Mutational Signatures

In the next step, we analyzed sample-specific mutational signatures to recognize the mutational processes playing a role in the mutagenesis of the analyzed BCC samples. Shortly, a mutational signature is a frequency pattern for different types of mutations (taking into account direct nucleotide context, -1 and +1 position) characteristic of particular cancer or cancer type. The pattern may reflect a main mutagenic process or a type of DNA repair deficiency that is specific to a given cancer. Originally based on analysis of single nucleotide variants, 30 distinctive mutational signatures were recognized in pancancer (36) but subsequently, the number of specific cancer signatures has been extended taking into account also other types of variants (53). The analysis showed that most of the samples were predominantly associated with signature 7 (average signature contribution (SC) = 0.7) and to a lesser extent with signature 11 (average SC = 0.2) (**Figures 1C, D**). Both signatures consist predominantly of C>T substitutions but differ in the sequence context of the substitutions. Signature 7 is associated with UV irradiation exposure and commonly occurs in melanoma and head and neck cancer. A hallmark of signature 7 is the frequent occurrence of double CC>TT substitutions resulting from UV radiation-induced pyrimidine dimers. Signature 11 was previously found in melanoma and glioblastoma multiforme, often in patients treated with the alkylating agent *temozolomide*, which is also used in BCC therapy. Only one sample (BCC22) showed a stronger association with signature 11 (SC = 0.6) than signature 7 (SC = 0.3). None of the analyzed samples showed an association with signatures 1, 2, 5, and 13, which are frequent in most cancer types. This may indicate that the deamination of 5-methylcytosine (5meC) predominantly induced by AID/APOBEC cytidine deaminases (attributed to the abovementioned signatures) does not play a role in the pathogenesis of BCC.

The comparison of the nodular and superficial BCC samples showed no substantial difference in terms of mutation burden or mutation types, with the exception of the contribution to mutational signature 7, which was higher for the nodular than superficial samples (**Figure 1E**), consistent with the higher UV radiation exposure of nodular BCCs.

### Hotspot Mutations

As recurrent mutations may be indicators of the cancer-related function of the mutated genes, we first looked for hotspots defined as genomic positions mutated in at least 3 samples (>10% of the cohort). In total, we identified 43 hotspots, including 23 hotspots in coding and 20 hotspots in noncoding regions (8 in 5’UTRs, 1 in 3’UTRs, and 11 in introns) (**Table S6**). Of the coding hotspots, 16 resulted in missense mutations, and 7 were synonymous substitutions. As the majority of synonymous mutations result from randomly occurring neutral alterations, we did not analyze the synonymous hotspot further. Although it has to be noted that the functionality of individual synonymous mutations cannot be unequivocally ruled out (51, 54, 55). For example, 315 (~2.1%) of the detected in our study synonymous mutations were predicted to be exonic splice-site mutations. Also, synonymous mutations located inside exons may affect different regulatory elements including exonic splicing enhancers and silencers (55). As shown in **Table S6**, some of the hotspots were located in genes annotated in the COSMIC Cancer Gene Census (CGC) database and/or in genes playing a role in cancer or skin function.

#### Hotspot Mutations in Coding Regions

Of the coding mutations (**Table S6**), the most commonly identified in our study (in 5 samples) was the c.1292C>T (Ser431Phe) substitution, located at chr14:103,131,144 in the Sec6 domain of *TNFAIP2*, which encodes a multifunctional protein playing a role in angiogenesis, inflammation, cell migration and invasion, cytoskeleton remodeling, and cell membrane protrusion formation (56–59). Nonetheless, *TNFAIP2* is not well-recognized in cancer, and the hotspot or other mutations in the gene have not been reported before. Another coding hotspot, mutated in 3 samples with the c.655C>T (Pro219Ser) substitution, was located at chr7:148,827,237 in *EZH2*; *EZH2* encodes an essential subunit (methyltransferase) of polycomb repressive complex 2 (PRC2), which plays a role in histone methylation and gene silencing (60)*. EZH2* is a well-known oncogene associated with a more aggressive form and poorer prognosis of many cancers, including melanoma, squamous cell carcinoma (SCC), and BCC, with demonstrated increased expression in SCC [compared to normal skin and SCC precursor actinic keratosis (AK)] (61) and aggressive BCC (62). Both gain- and loss-of-function mutations in *EZH2* have often been found in myeloid leukemias and lymphomas but are not common in solid tumors. Contrary to the previously detected mutations clustering mostly in the catalytic SET domain (63, 64), the hotspot detected here was located in the N-terminal (NT) part of the protein, which, among other areas, is responsible for interaction with histones (65). Whether the mutations may affect the interaction warrants further investigation. To the best of our knowledge, this mutation hotspot has not been observed in any cancer, including BCC.

An additional interesting coding hotspot (mutated in 3 samples) was located at chr15:40,382,906-40,382,907. The hotspot was mutated with either the c.71C>T substitution or the c.71_72delinsTT double substitution (note that double substitutions are annotated as deletion/insertion (delins) variants according to HGVS nomenclature), both resulting in the Ser24Phe missense mutation affecting the NT part of the KNSTRN protein [also known as small kinetochore-associated protein (SKAP)], which plays a role in maintaining chromatid cohesion and proper chromatid separation during anaphase (66). *KNSTRN* mutations (predominately the Ser24Phe hotspot mutation) were first detected in 19% of SCCs and 13% of AKs (67). Subsequent analysis of The Cancer Genome Atlas (TCGA) datasets showed that the *KNSTRN* mutations also occur in 5% of melanoma samples but are rare in other cancers. Later, *KNSTRN* mutations were also identified in 2% (15) and 10% (68) of BCCs. These findings together with this study confirm that *KNSTRN* mutations are specific to UV radiation-related skin cancers. Consistent with the role of KNSTRN, it was shown that *KNSTRN* mutations in SCC affect proper chromosome separation and are associated with increased chromosome instability, expressed as a fraction of the genome with copy number alterations (CNAs) (67). Although there was a similar number of tested samples, the association of the KNSTRN mutations with CNAs was not confirmed in BCC, neither in a study by Jaju et al. (68) nor in our study (**Figure S1**). It is worth noting that it was also shown that KNSTRN plays a role in UV radiation-induced apoptosis (69); however, the effect of the mutations on avoidance of apoptosis by BCC cells or any other cancer cells has not yet been tested.

#### Hotspot Mutations in Noncoding Regions

**Figure 2:**
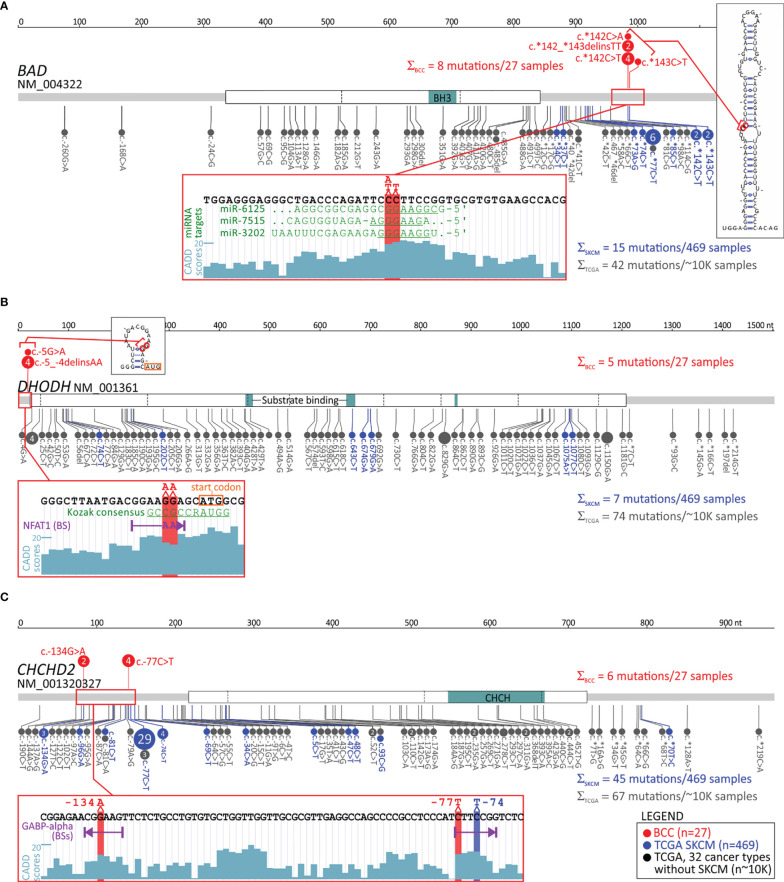
Distribution of mutations in the selected genes with the identified mutation hotspots in noncoding areas. **(A-C)** Maps of the *BAD*, *DHODH*, and *CHCHD2* genes, with the exon structure and protein functional domains indicated. Mutations are visualized in the form of lollipop plots along with the gene maps, and the size of a mutation symbol (circle) is proportional to the number of mutations. Mutations identified in BCC (red) are shown above and mutations identified in SKCM (blue) and other TCGA cancers (gray) are shown below the maps. The inset below each map shows the detailed sequence context of the hotspot mutations, along with CADD score graphs, indicating the functional relevance of particular positions and other sequence characteristics (i.e., (in **A**) predicted miRNA target sites, (in **B**) the Kozak consensus sequence and NFAT1 transcription factor binding sites (BSs) created by the hotspot mutation, and (in **C**) the GABP-alpha transcription factor BSs disrupted by the hotspot mutations). The additional insets in **(A, B)** show computationally predicted RNA secondary structures generated from RNA sequences directly flanking the hotspots. * represents stop codon.

The most frequently mutated hotspot of all the hotspots detected in the study (mutated in 8 samples) was located at chr11:64,270,066-64,270,067 in the 3’UTR of *BAD* and has never been reported before. The hotspot encompasses 4 different substitutions (c.*142C>A, c.*142C>T, c.*142_*143delinsTT and c.*143C>T; **Table S6** and **Figure 2A**), located 142 or 143 nucleotides (nt) downstream of the stop codon. The protein encoded by the gene is a member of the BCL-2 family, which plays a role in the positive regulation of cell apoptosis. The gene is commonly implicated in many cancers (70, 71); however, to the best of our knowledge, this hotspot has not been reported before in any cancer.

Next, another novel noncoding hotspot mutated in 5 samples located at chr16:72,008,760-72,008,761 in the 5’UTR of *DHODH* was identified. The hotspot encompasses two different substitutions, c.-5G>A and c.-5_-4delinsAA, affecting the Kozak sequence (**Table S6** and **Figure 2B**). *DHODH* is not well studied in cancer, but it has recently been demonstrated that it plays an important role in the carcinogenesis of SCC and other UV radiation-induced skin cancers (72, 73).

Another mutated noncoding hotspot from our study worth mentioning was found in 4 samples with the c.-77C>T substitution and was located at chr7:56,106,490 in the 5’UTR of *CHCHD2*, also known as *MNRR1* (**Table S6** and **Figure 2C**). The analysis of the entire *CHCHD2* 5’UTR showed one more recurrent (in 2 samples) substitution, c.-134G>A, located at chr7:56,106,547, resulting in a total of 6 mutations in the 5’UTR in 6 samples. Interestingly, frequent mutations in the hotspot in the 5’UTR of *CHCHD2* were previously reported in melanoma (74).

Finally, we identified a hotspot located at chr1:153,990,763 in the 5’UTR of *RPS27* (encoding a ribosomal protein component of the 40S subunit) that was mutated in 3 samples with the c.-34C>T substitution. Mutations in the promoter/5’UTR of *RPS27* (including the hotspot mutation) have been identified before in ~10% of melanoma samples (74, 75) but have never been reported in BCC or other skin cancers. Subsequent *in vitro* functional studies showed that the *RPS27* 5’UTR hotspot mutation decreases *RPS27* mRNA levels and that decreased levels of RPS27 are associated with a worse prognosis of melanoma patients and drug (*vemurafenib* and *palbociclib*) sensitivity of melanoma cells (76).

#### Computational Analysis of the Identified Noncoding Hotspots and Comparison With External Datasets

To further characterize three noncoding hotspot mutations, two not previously reported in *BAD* and *DHODH* and one in *CHCHD2* previously reported in melanoma (74), we analyzed their potential impact with a number of computational tools and investigated their incidence in other cancers using external datasets of a large cohort (>10,000 samples) of TCGA samples, representing 33 different human cancer types (including 469 skin cutaneous melanoma (SKCM) samples but not including BCC or SCC samples). Note that the list and the standard abbreviations of all TCGA cancer types are in **Table S3**.

In total, in the TCGA samples, we identified 28 mutations in the *BAD* 3’UTR (**Figure 2A**). The mutations were found predominantly in SKCM samples (15 mutations in 12 (2.6%) SKCM samples), including 4 mutations in the hotspot (residues c.*142C and c.*143C) identified in BCC, and 6 c.*77C>T mutations, constituting an additional hotspot in the 3’UTR, not occurring in BCC. In other cancers, 3’UTR mutations were very rare (**Figure 2A**). In contrast with the mutation frequency in the 3’UTR, mutations in other parts of the gene, including the coding region (n=26, predominantly missense or synonymous), were rare (not exceeding 1% in any cancer) and randomly distributed between different cancer types (excluding SKCM). The exclusiveness of the SKCM and BCC mutations in the 3’UTR *vs*. other parts of the gene (enrichment compared to other cancer types; Fisher’s exact test; p<0.0001 and p=0.0005, respectively) precludes an accidental occurrence of the mutations, solely as a result of some region- and/or mutagenesis-related mechanisms and argues for the cancer-driven selection of the 3’UTR mutations in BCC and SKCM (and likely also in other UV irradiation-related cancers).

Next, with the use of TargetScan, we identified 3 miRNAs (miR-7515, miR-3202, and miR-6125) whose predicted targets (seed-interacting sequences) were disrupted by hotspot mutations (**Figure 2A**). However, as (i) none of these targets has been validated by any means [miRTarBase (77)], (ii) none of these miRNAs have been confidently validated (*via* miRBase or miRGeneDB), and (iii) none of these miRNAs have been found to have expression levels detectable/confirmed in any of the TCGA cancers, it is very unlikely that any of the identified targets are functional. Additionally, the occurrence of SKCM mutations in different positions across the *BAD* 3’UTR argues against the possibility that the driving force of the mutations is a disruption of a particular miRNA target. Some clue for the functionality of the BCC hotspot may be its location in the 5’ arm of the ~40 bp long stable hairpin RNA structure motif (dG=-39.6 Kcal/mol), which is destabilized (by ~2 Kcal/mol) by the hotspot mutations (**Figure 2A**).

The analysis of TCGA data showed no mutation in the BCC hotspot or any other mutation in the *DHODH* 5’UTR in any of the TCGA cancer types, even though different mutations (n=81) were identified in other parts of the gene, including 75 mutations in the coding region (**Figure 2B**). The other mutations, however, were randomly distributed along the gene sequence and between different cancer types, and only two of the coding mutations were deleterious (frameshift) mutations. This result indicates that the *DHODH* 5’UTR hotspot mutations are BCC-specific mutations, and the absence of these mutations in other UV radiation-related cancers makes it unlikely that the frequent occurrence of the mutations in BCC is solely due to a random effect of UV irradiation. The 5’UTR of *DHODH* is very short (21 bp). Although hotspot mutations occurred in the Kozak sequence, which is important for the initiation of translation, neither wild-type nor mutant alleles affected the consensus Kozak sequence nucleotides (at positions -4 and -5); therefore, the ATGpr (78)), and NetStart 1.0 (GedersenAG (79) tools predicted the mutations to have a minor effect on the effectiveness of translation under standard conditions. However, this result does not exclude an effect of the mutations under specific conditions, such as hypoxia, UV exposure, or cancer.

The analysis of RNA secondary structure showed that the hotspot mutations slightly modified (decreased the stability of) a small hairpin motif predicted to be formed by an RNA sequence directly flanking the hotspot (**Figure 2B**). The mutation may also destabilize the potential long-range interaction of the sequence flanking the mutations with the sequence located ~200 nt downstream. Analysis of the 5’UTR sequence (80) showed that the double substitution (GG>AA) at the hotspot creates a consensus binding site for the NFAT1 transcription factor (**Figure 2B**), which is expressed in many tissues, including sun-exposed and non-sun-exposed skin (GTExPortal; GTEx Consortium Science 2020), and implicated in many cancers, including melanoma (81, 82).

In total, in TCGA data, we identified 63 mutations in the *CHCHD2* 5’UTR (**Figure 2C**). The mutations were found predominantly in SKCM samples (40 mutations in 39 (8.5%) samples), including 29 c.-77C>T mutations and 3 c.-134G>A mutations, located in the hotspot positions identified in BCC. Additionally, we identified 4 samples with the c.-74C>T mutation, constituting an additional hotspot in the 5’UTR. Only 5 SKCM mutations were located outside the 5’UTR, 4 in the CDS (2 missense and 2 synonymous), and 1 in the 3’UTR (one mutation) (**Figure 2C**). In other cancers, there were rare 5’UTR mutations, including 4 mutations in HNSC and UCEC, 3 mutations in BRCA, and 12 mutations in other cancers. Three of these mutations coincided with the c.-77 hotspot. The positions of BCC/SKCM hotspot mutations seem to be nonrandom because they were all located in and all disrupted two distinct GABP-alpha transcription factor binding sites [mapped with the use of MotifMap (50)] (**Figure 2C**).

### Frequently Mutated Genes

Next, we looked at the overall frequency of mutations in the genes, separately analyzing mutations in coding regions, 5’UTRs, 3’UTRs, and introns (defined in Materials and Methods; listed in **Table S7**). Although they were not considered frequently mutated, in this section, we also report genes with any mutations in a coding region if they were detected in a pathway of a recurrently mutated gene. In the analysis of frequently mutated regions, we focused mostly on genes functionally related to cancer (annotated with CGC and a manual literature search) and genes playing a role in skin function.

#### Genes Frequently Mutated in Coding Regions

**Figure 3:**
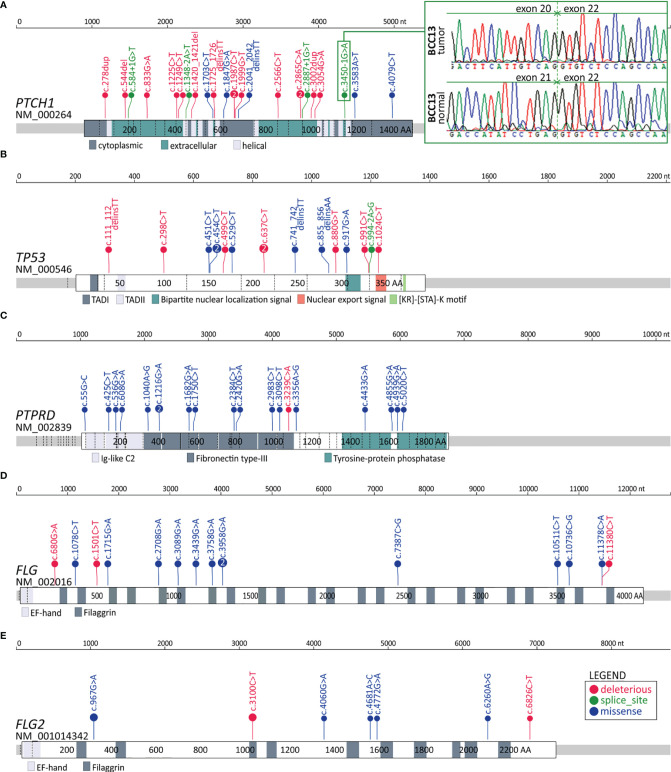
Distribution of the identified mutations in the genes with frequent mutations in the coding sequence. **(A-E)** Maps of the *PTCH1*, *TP53*, *PTPRD*, *FLG*, and *FLG2* genes. Mutations are visualized in the form of lollipop plots along with gene maps; the size of a mutation symbol (circle) is proportional to the number of mutations, and the color indicates the type of mutation (as shown in the legend). Additionally, the inset in **(A)** shows the Sanger sequencing reads depicting the effect of the splice-site mutation c.3450-1G>A on exon 21 skipping.

**Figure 4:**
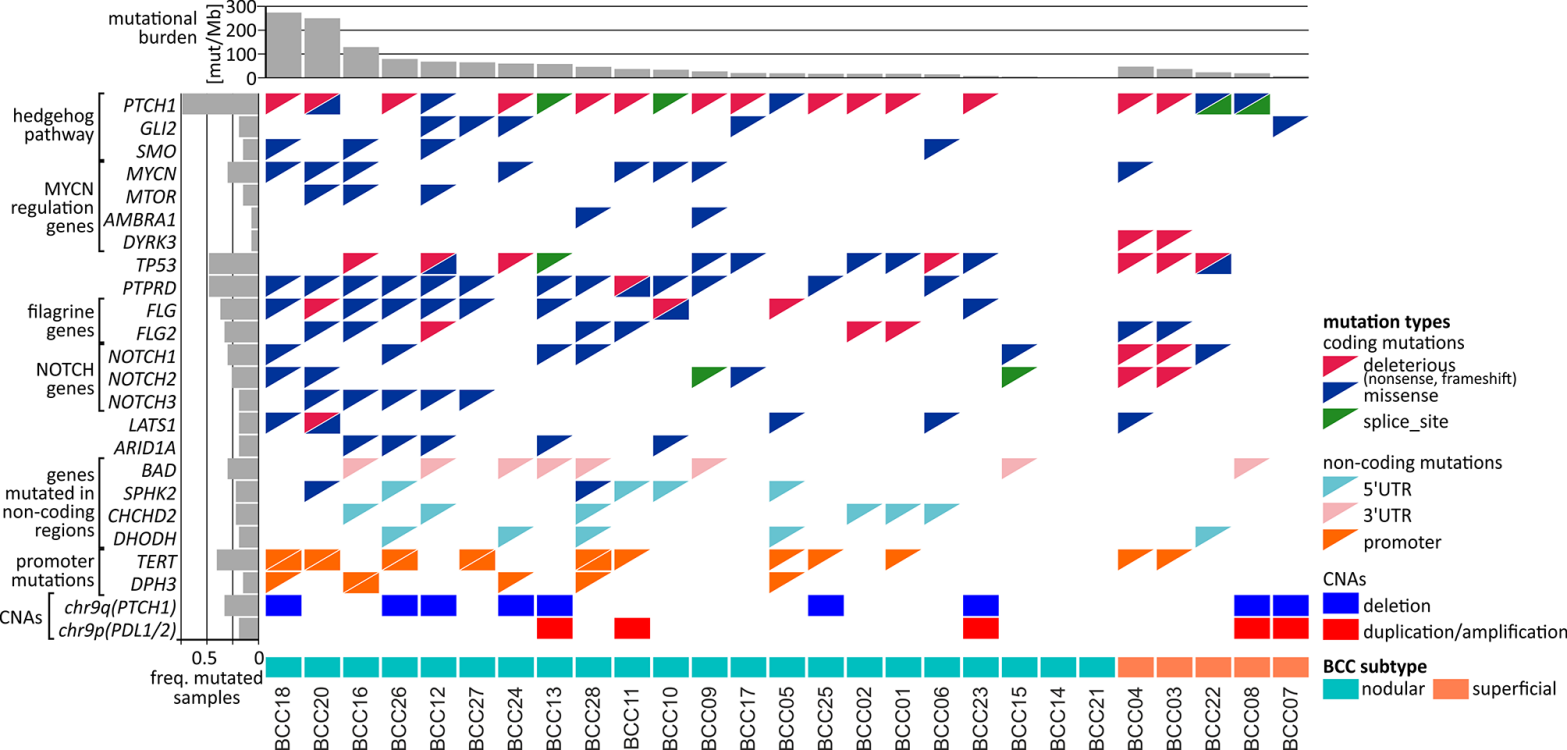
Comutation plot summarizing the somatic alterations in the BCC samples. Columns correspond to the samples, and rows correspond to the selected genes. The color of the mutation presence symbols corresponds to the mutation type, as indicated in the legend on the right. The bar plots above and on the left indicate the mutational burden and the fraction of samples with mutations in particular genes, respectively. The nodular and superficial samples are indicated by color.

In total, we identified 606 genes frequently mutated in coding regions. The most frequently mutated was *PTCH1*, with a total of 24 mutations in 20 BCC samples, including 5 missense, 4 splice-site, and 15 deleterious (nonsense or frameshift) mutations (**Figure 3A**). Mutation c.3450-1G>A located upstream of exon 21 was one of the splice-site mutations and was also observed in another study (14), which suggests its recurrence in BCC. We tested and confirmed the exon-skipping effect of the mutation with the use of exon-junction PCR and Sanger sequencing analysis (**Figure 3A**). The other genes from the hedgehog pathway recurrently mutated in our cohort were *GLI2*, which was mutated in 5 samples, and *SMO*, which was mutated in 4 samples (**Figure S4** and **Figure 4**). The combined frequency of *SMO* and *GLI2* mutations was much lower in samples with (4/20; 20%) than in those without (4/7; 57%) *PTCH1* mutations, which suggests mutual exclusiveness of these mutations (**Figure 4**). Altogether, 24 (88%) samples had mutations in genes involved in the hedgehog pathway. Other frequently mutated cancer-related genes were *TP53* (7 missense, 8 deleterious, and one splice-site mutation in 13 samples) (**Figure 3B**); *MYCN* (8 missense mutations in 8 samples), *NOTCH1* (8 missense and 2 deleterious mutations in 8 samples), *NOTCH2* (3 missense, 3 deleterious, and 2 splice-site mutations in 7 samples), *NOTCH3* (6 missense mutations in 5 samples; note that the *NOTCH* mutations colocalized with the regions of the loss-of-function mutations identified in other solid tumors, e.g., in SCCs (83), *LATS1* (5 missense and one deleterious mutation in 5 samples), and *ARID1A* (5 missense mutations in 5 samples) (**Figure 4** and **Figure S2**). The mutations in the abovementioned genes are generally consistent with mutations observed before in BCC (14, 15). Additionally, we identified very frequent mutations (18 missense and 1 deleterious) in *PTPRD* (**Figure 3C**), a tumor suppressor frequently mutated in many cancers, including melanoma and cutaneous SCC (84–88), in 13 samples, but these have never been reported as frequently mutated in BCC.

Interestingly, in addition to mutations in *MYCN*, we also noticed recurrent (although not frequent) mutations in three other genes in the MYC/MTOR regulatory network, i.e., *MTOR*, *DYRK3*, and *AMBRA1* (**Figure 4**), which have not been reported as mutated in BCC. The *MTOR* missense/activating mutations identified in other cancers are considered biomarkers for therapy with mTOR pathway inhibitors (89).

Finally, we found a high frequency of mutations in the *FLG* (15 mutations in 10 samples) and *FLG2* (9 mutations in 9 samples) genes (**Figures 3D, E** and **Figure 4**), encoding profilaggrin and filaggrin-like proteins, precursors of filaggrin. Filaggrin is an important component of the stratum corneum of the epidermis that plays a role in maintaining epithelial homeostasis and barrier functions (90) and is a substrate for trans-urocanic acid (UCA) and pyrrolidone carboxylic acid (PCA), which are suggested to serve as a natural UV radiation barrier (91). Although frequent mutations in the *FLG*/*FLG2* genes have been previously observed in other cancers, the mutations were usually considered random (passenger). Here, however, we observed a relatively high proportion of deleterious nonsense mutations, altogether occurring in 6 samples. Additionally, the analysis of the entire cohort of TCGA samples showed that the frequency of the *FLG*/*FLG2* mutations observed in our study in BCC substantially exceeds the frequencies of the mutations in other cancers, including melanoma (the next most frequently mutated cancer) (**Figure S3**).

#### Genes Frequently Mutated in Noncoding Regions

Among the 11 genes frequently mutated in the 5’UTR (**Table S7**) there were *DHODH* and *CHCHD2* with the hotspot mutations described above (see subsection Hotspot mutations). Of interest may also be *SPHK2*, with 4 dispersed mutations in 4 samples, whose function as both a proapoptotic gene suppressing cell growth and an oncogene promoting cell proliferation has been proposed (92–96). *SPHK2* also had mutations in its coding region (**Figure 4**).

Among the 11 genes frequently mutated in the 3’UTR (**Table S7**), in addition to BAD described above (see subsection Hotspot mutations), we also identified 8 mutations in the 3’UTR of *SMIM27* (also annotated as lncRNA *TOPORS-AS1*); the overexpression of *SMIM27* was found to be associated with favorable outcomes in breast cancer (97).

Finally, we identified 289 genes (15 annotated in CGC) frequently mutated in introns (**Table S7**). Interestingly, among the genes was *PTCH1*, which, in addition to 4 splice-site mutations (mentioned above), also had other 4 intronic mutations (in total, 8 intronic mutations). Other genes with frequent mutations in introns included *PTPRD* (14 mutations in 9 samples), which also frequently had mutations in the coding region; *NOTCH2* (6 mutations, including 2 splice-site mutations in 6 samples), which also frequently had mutations in the coding region; *ERBB4* (6 mutations in 6 samples), a well-known oncogene playing a role in many cancers [reviewed in (98)]; and *DROSHA* (5 mutations in 5 samples), which encodes a core enzyme (nuclease) of the miRNA processing pathway and has been shown to be upregulated in BCC (99).

#### Mutations in the *TERT* and *DPH3* Promoters

The only noncoding mutations previously studied in BCC are mutations recurrently occurring in promoters of *TERT* and *DPH3* (27, 100, 101). As these promoters were not covered in our exome sequencing experiment, we performed Sanger sequencing for these regions. As a result, we have detected 16 mutations in 11 (41%) patients in the *TERT* promoter and 6 mutations in 5 (19%) patients in the *DPH3* promoter (**Figure 4**). All *TERT* mutations were detected in previously described positions and well-known hotspots responsible for the recruitment of transcription factors activating expression of *TERT* in cancer, including 2 double substitutions c.-139_-138delinsAA, 9 substitutions c.-146G>A, 2 substitutions c.-101G>A, and 3 other substitutions (c.-150G>A, c.-100G>A, and c.-99G>A). Also, *DPH3* mutations were located in positions described before (27, 102), including 3 double substitutions c.-122_-121delinsTT, and 3 other substitutions (c.-150C>T, c.-122C>T, and c.-121C>T).

### Driver Genes in BCC (OncodriveFML Analysis)

**Figure 5:**
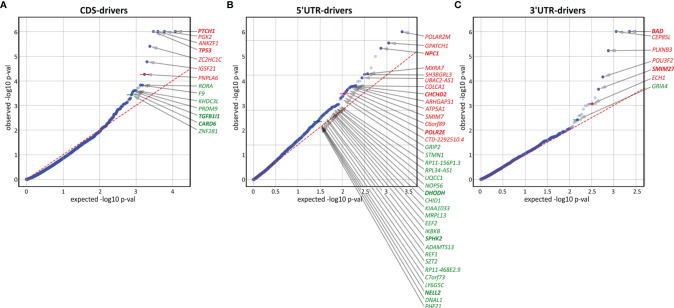
Identification of potential cancer drivers with the use of OncodriveFML. The quantile-quantile (QQ) plots show the distribution of expected (x-axis) and observed (y-axis) p-values corresponding to FM bias calculated (with CADD score) separately for mutations in **(A)** coding regions, **(B)** 5’UTRs, and **(C)** 3’UTRs. The green and red colors indicate genes defined as significant (q<0.025) and highly significant (q<0.01), respectively, according to OncodriveFML recommendation.

To further investigate the mutations/mutated genes, we used OncodriveFML, which allows the prediction of the cancer driver potential of both coding and noncoding regions/genes based on functional mutation (FM) bias (39). As shown in **Figures 5A–C** and **Table S8**, we identified 14 potential cancer driver genes based on mutations in coding regions (CDS-drivers), a disproportionately high number of 36 potential cancer driver genes based on mutations in 5’UTRs (5’UTR-drivers), and 7 potential cancer driver genes based on mutations in 3’UTRs (3’UTR-drivers). No potential cancer driver gene was identified based on the mutations in introns.

In addition to 4 CDS-drivers (*PTCH1*, *TP53*, *TGFB1I1*, and *CARD6*) also identified as frequently mutated, it is worth noting *RORA*, recently shown to play an important role in restraining allergic skin inflammation (103). Other interesting genes were *PRDM9* and *ZNF281*, both of which play a role in DNA repair and have been shown to be responsible for frequent mutations in cancer (104, 105). None of these genes were previously implicated or identified as frequently mutated in BCC.

Among the 5’UTR-drivers, 6 were also identified as frequently mutated: *DHODH*, *CHCHD2*, and *SPHK2* (described above), as well as *POLR2M*, *NPC1*, and *NELL2*. Additionally, it is worth noting *IKBKB* (mutated in 3 samples but not reported before as mutated in BCC) shown to act as a tumor suppressor in nonmelanoma skin cancers and noncancerous skin lesions; it was also shown that deletions of the gene lead to skin inflammation, hair follicle disruption, hyperplasia, and SCC development (106–109).

Among 3’UTR-drivers, two genes (mentioned above), i.e., *BAD* (the most significant 3’UTR-driver) and *SMIM27* were also identified as frequently mutated. Additionally, it is worth mentioning the transcription factor gene *POU3F2* (mutated in 3 samples), that plays a role in the invasiveness and metastasis of melanoma, and is controlled by miR-211 (110, 111) and miR-107 (112). Although the mutations were not located in the predicted miR-107 and miR-211 binding sites, they may affect the structure of the 3’UTR and thus indirectly change accessibility to these or other miRNA targets.

### Analysis of Copy Number Alterations

**Figure 6:**
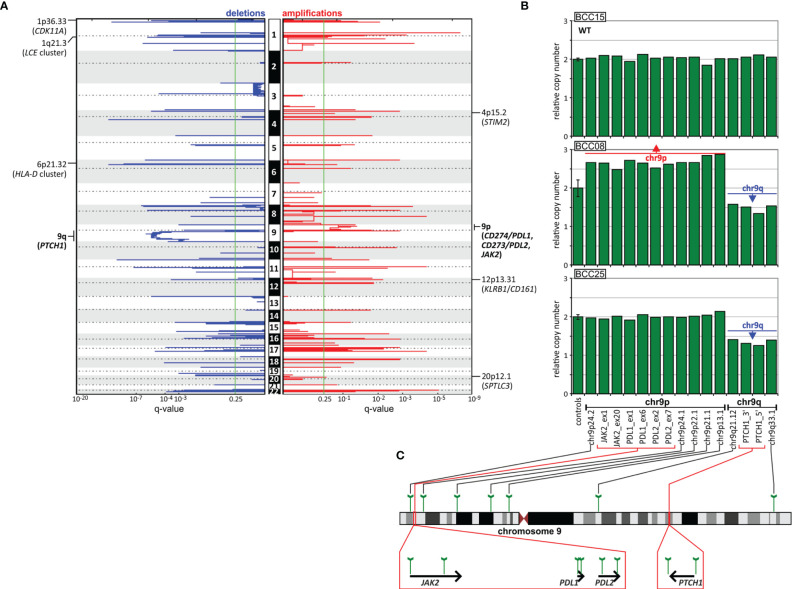
CNA analysis of the BCC samples. **(A)** GISTIC-estimated q-values for deletions (left, blue) and amplifications (right, red) are plotted along with chromosome positions (vertically). The green line indicates the recommended significance threshold, q=0.25. The selected significantly deleted and amplified regions/genes are indicated on the graphs. **(B)** Representative MLPA results (bar plots), showing samples with chromosome 9 CNAs, i.e., chr9q deletion and chr9p amplification, *vs*. a sample (at the top) with the wild-type (WT) copy number genotype. Each bar plot depicts relative copy number values (y-axis) of the probes specific for regions along chromosome 9 and an average (with standard deviation error bar) signal of control probes (x-axis). **(C)** Schematic depictions of the localization of the probes on chromosome 9 and in genes of interest.

As somatic CNAs have not been extensively studied in BCC, in the next step, we performed analysis of both chromosome arm-level and focal CNAs [with GISTIC2 (40)]. At the chromosome arm level, we detected a significant recurring deletion of chr9q (q=1.4x10-6; occurring in 9 samples), involving *PTCH1* (**Figures 4**, **6**), and a significant recurring amplification of chr9p (q=0.05; occurring in 5 samples), involving a region with *CD274* (also known as *PDL1*, encoding PD-L1), *CD273* (also known as *PDL2*, encoding PD-L2), and *JAK2* (**Figures 4**, **6**). Although the loss of chr9q has been frequently observed in BCC (reported as loss-of-heterozygosity of *PTCH1*), gain of chr9p has been reported only in one case of rare metastatic BCC (113). To validate the chromosome 9 CNAs, we developed a multiplex ligation-dependent probe amplification (MLPA) assay with probes covering the entire chromosome 9 but especially focusing on the region containing *PTCH1* (chr9q22.32) and the region harboring *PDL1*, *PDL2*, and *JAK2* (chr9p24.1) (**Figure 6**). The MLPA analysis confirmed CNAs in all tested samples as detected by GISTIC2, and examples are shown in **Figure 6**.

CNA analysis also showed 54 regions of significant focal deletions, including 27 regions containing skin/cancer-related genes, and 56 significant amplifications, including 20 encompassing skin/cancer-related genes (**Figure 6** and **Table S9**). The elements involved in the most significant focal deletions were *CDK11A* (chr1p36.33; q=2.4x10-5; occurring in 6 samples), whose loss induces skin carcinogenesis (114); the *LCE* cluster (chr1q21.3; q=2.4x10-6; occurring in 4 samples), including genes such as *LCE2* and *LCE3*, which play a role in maintaining skin barrier function and whose deletion has been associated with psoriasis (115); and the *HLA-D* cluster (*HLA-DP*, -*DQ*, and *-DR*, chr6p21.32; q=2x10-4; occurring in 3 samples), encoding components of major histocompatibility complex (MHC) class II molecules, whose increased expression has been associated with increased cancer immunogenicity and better prognosis in BCC, SCC and melanoma (116–122). The skin/cancer-related genes in the most significant focally amplified regions worth mentioning are *STIM2* (chr4p15.2; q=0.16; occurring in 2 samples) (123), *KLRB1/CD161* (chr12p13.31; q=0.007; occurring in 2 samples) (124, 125), and *SPTLC3* (chr20p12.1 q=0.23; occurring in 2 samples) (126).

## Discussion

In this study, we detected thousands of mutations in BCC samples, many of which were clustered in specific genes/regions or hotspots located in both coding and noncoding regions. Despite the small size of our dataset, our results are in line with those of previous genomic analyses of coding mutations in BCC (14, 15), which confirms the reliability of our study. We believe that our results may give valuable insights related to general characteristics of mutations such as mutational burden or mutational signatures and in terms of genes identified as recurrently mutated in coding regions.

Moreover, we extended our analysis to noncoding parts of the genes, which altogether were responsible for ~50% of the mutations identified by the standard WES approach. Variants in such areas have usually been ignored in previous BCC genetic studies. Many of the identified noncoding hotspots were located in sequences of genes functionally related to cancer or more specifically to UV radiation-related skin cancers. Some of them were reported before in melanoma or identified by us in melanoma TCGA samples, the cancer type most intensively studied in terms of mutations in noncoding regions (127, 128). Below, we briefly describe the cancer-related role of the three most interesting genes with hotspot mutations in noncoding regions, i.e., *BAD*, *DHODH*, and *CHCHD2*. Interestingly, all these genes have functions related to mitochondrial activity.

Of all the hotspots detected in our study, the most frequently mutated was the hotspot located in the 3’UTR of *BAD*. This hotspot had several different mutations affecting 2 nucleotide positions (142 and 143 nt downstream of the stop codon). Due to these mutations, *BAD* was also classified as being highly mutated in the 3’UTR and as the top most significant potential cancer driver. Consistently, the hotspot and several other positions in the 3’UTR are frequently mutated in melanoma but not in other cancers. BAD belongs to the BCL-2 family, consisting of both proapoptotic and antiapoptotic proteins. It promotes cell death by inducing mitochondrial outer membrane permeabilization (MOMP), allowing the release of cytochrome c, and by antagonizing (dimerizing with) antiapoptotic BCL-2 proteins (129, 130). On the other hand, phosphorylated BAD may also have antiapoptotic properties, e.g., promoting the survival of melanocytes (131, 132). Other functions of BAD include regulation of mitochondrial metabolism (regulation of voltage-dependent anion channels and metabolite passage through the outer mitochondrial membrane) and dynamics (regulation of shape changes) (133–139). Although BAD has not been previously implicated in skin cancers, loss or downregulation of other proapoptotic members of the BCL-2 family, i.e., BAX and PUMA, has been shown to promote the development of BCC, SCC, and cutaneous melanoma (140, 141). Therefore, a similar effect may be induced by mutations causing more efficient downregulation of BAD.

*CHCHD2* is a gene with frequent mutations in the 5’UTR, the hotspot mutation c.-77C>T and the recurrent mutation c.-134G>A (77 and 134 upstream of the start codon). Based on the 5’UTR mutations, *CHCHD2* was classified as a high-priority cancer driver. We showed that the *CHCHD2* 5’UTR (predominantly the hotspot position) was also frequently mutated (8%) in the SKCM TCGA samples, which also showed the additional recurrent mutation c.-74C>T. The 5’UTR mutations were also found in whole-genome sequenced Australian melanoma samples (74). The role of the gene has not been intensively studied in cancer, but it was shown that under hypoxic conditions, CHCHD2 is translocated from the mitochondrial intermembrane space to the nucleus, where it binds an oxygen-responsive element in the promoter of *cytochrome oxidase 4I2* (*COX4I2*), encoding a subunit of complex IV of the electron transport chain, and increases its expression. Consequently, *CHCHD2* knockdown downregulates *COX4I2* and decreases cell oxygen consumption (142). It was also shown that CHCHD2 is a negative regulator of mitochondria-mediated apoptosis (143). Liu et al. showed that CHCHD2 interacts with antiapoptotic BCL-XL (from the BCL-2 family), which leads to inhibition of proapoptotic BAX and consequently decreases MOMP and apoptosis. In addition, it was shown that CHCHD2 dysregulates multiple genes that play a role in cell migration and cancer metastasis and that its expression is higher in cell lines derived from more aggressive breast tumors (144). Consistent with the function of CHCHD2 related to mitochondrial metabolism, we found that all BCC/SKCM hotspot/recurrent mutations coincided with and impaired two distinct binding sites of GABP-alpha. As GABP-alpha is known to be a transcription factor involved in the regulation of cellular energy metabolism and cell cycle regulation (145), this finding might hint at a functional role of the mutations in cancer. Of note, germline missense mutations in *CHCHD2* are associated with autosomal dominant Parkinson’s disease (146).

*DHODH* is a gene that showed frequent mutations in the Kozak sequence of the 5’UTR, with hotspot mutations encompassing two different substitutions, c.-5G>A and c.-5_-4delinsAA (4 and 5 nt upstream of the start codon). Based on the identified mutations, *DHODH* was classified as a candidate cancer driver. The analysis of the entire TCGA cohort (~10K samples from 33 cancer types) showed that no other cancer had mutations in the hotspot or the 5’UTR, indicating that the mutations were BCC-specific. Although *DHODH* 5’UTR mutations have never been reported before in any cancer, it was shown very recently that *DHODH* plays a key role in the carcinogenesis of SCC and other UV radiation-induced skin cancers and facilitates the development of precancerous skin lesions (72, 73). Hosseini et al. showed that the DHODH protein level and enzymatic activity are markedly upregulated in irradiated skin and that an increased level of DHODH sensitizes the skin to UV irradiation-induced damage. It was also shown that DHODH is upregulated in melanoma, in which DHODH inhibition leads to a marked decrease in tumor growth both *in vitro* and in mouse xenograft studies (147). DHODH inactivation inhibits cell proliferation and induces cell cycle arrest at the S phase in BCL-2 (pro-apoptotic) deficient melanoma cells (148). DHODH is embedded in the inner mitochondrial membrane, and its canonical role is in the oxidation of dihydroorotate to orotate, an important step in *de novo* pyrimidine synthesis (which is important in replication and DNA repair). However, a side product of the pathway, ubiquinol (QH2), is a source of electrons in the electron transport chain, and DHODH also plays a role in alternative (glucose-independent) respiration (utilizing amino acids as an energy source) (72, 73, 148), facilitating cancer development in hypoxic conditions. In addition, it was found that in esophageal SCC, elevated DHODH levels promote cell proliferation by stabilizing β-catenin (149). The functional effects of the mutations may result from alteration of the Kozak sequence but also the creation of an NFAT1 transcription factor binding site, which is not present in the wild-type sequence. NFAT1 is a widely distributed isoform of the NFAT family of transcription factors and is expressed in tumor cells and the tumor microenvironment (150). The constitutive activation and overexpression of NFAT1 in many cancer types promote the transcription of genes that are crucial for cancer development and progression, including *COX2*, *MMP7*, *MMP9*, and *MDM2* (151, 152).

It is worth noting that the only noncoding mutations analyzed in BCC before are the mutations in promoters of *TERT* and *DPH3* (27, 100, 101); which are known to be mutated in many cancers, including melanoma (127, 128). Although our WES design generally did not cover promoter regions, with the use of Sanger sequencing, we confirmed high frequency and high recurrence of promoter mutations in *TERT* (41% of patients) and *DPH3* (19%).

Additionally, the whole-genome CNA analysis allowed us to detect two highly significant chromosome-level CNAs. In addition to the expected deletion of chr9q, consistent with the loss of heterozygosity of *PTCH1*, we also detected frequent duplication/amplification of chr9p, encompassing the *PDL1* and *PDL2* genes (which encode the two immune checkpoint proteins PD-L1 and PD-L2, the overexpression of which enables cancer cells to evade the host immune system). Copy number gains of *PDL1* have been observed only in one case of metastatic BCC (113). The patient, who was otherwise resistant to *vismodegib* and *sonidegib*, demonstrated a dramatic response to *nivolumab* (an anti-PD-1 antibody blocking the PD-1/PD-L1 interaction), which strongly suggested that the copy number gain may be a biomarker of sensitivity to anti-PD-1/PD-L1 checkpoint treatments (113). It was also shown in an independent study that some patients (up to ~40%) with advanced BCC (not tested for *PDL1* amplification) respond to *pembrolizumab* (another anti-PD-1 antibody) (113, 153). Therefore, assessment of copy number gains of the *PDL1/PDL2* region may help to rationalize such treatment; however, further study with a larger group of samples is required.

Finally, we would like to note the apparent limitations of the study. As it was intended to be a preliminary evaluation of noncoding mutations in BCC, we analyzed only a small number of samples, and as such, we limited the characterization of the identified variants to computational analyses. It has to be also noted that our analysis covered only a small fraction (~1%) of the noncoding genome that cumulatively accounts for ~98% of the genome and contains many different functional elements not covered in our analysis, including promoters, enhancers, and genes of different classes of non-coding RNAs.

In summary, in this study utilizing WES BCC data, we revealed not only mutations in coding regions of previously known BCC-related genes but also frequent mutations in noncoding regions of cancer-related genes, some of which may be strong candidates for new BCC drivers. Although the functional role of the individual identified genes/mutations requires further experimental interrogations, our results provide a strong basis for further analyses of noncoding variants in BCC and other cancer types.

## Data Availability Statement

The datasets presented in this study can be found in online repositories. The names of the repository/repositories and accession number(s) can be found below: https://www.ncbi.nlm.nih.gov/, PRJNA747148.

## Ethics Statement 

The studies involving human participants were reviewed and approved by bioethics committee of the Ruhr-University of Bochum. The patients/participants provided their written informed consent to participate in this study.

## Author Contributions

PN extracted the DNA samples, performed almost all experimental and computational analyses, drafted the manuscript (with PK), prepared all figures, tables, and supplementary materials. PG-M participated in the study design, extracted mutations from TCGA, assisted PN in experimental analyses, participated in the manuscript preparation. MU-T prepared the scripts for mutation calling and mutations annotation, assisted PN in computational analyses, participated in the manuscript preparation. IM-T performed the analysis of the NFAT1 TF binding site in the promoter of DHODH, participated in the manuscript preparation. NS participated in CNA analysis with GISTIC2, participated in the manuscript preparation. AP assisted in computational analyses, participated in the manuscript preparation. LS – collected and characterized the BCC samples, participated in the manuscript preparation. MS – participated in conceiving the study, participated in collection and characterization of BCC samples, participated in the manuscript preparation. PK – conceived and supervised the study, drafted the manuscript (with PN), acquired the funding. All authors contributed to the article and approved the submitted version.

## Funding

This work was supported by research grants from the Polish National Science Centre [2016/22/A/NZ2/00184 and 2020/39/B/NZ5/01970] (to PK).

## Conflict of Interest

The authors declare that the research was conducted in the absence of any commercial or financial relationships that could be construed as a potential conflict of interest.

## Publisher’s Note

All claims expressed in this article are solely those of the authors and do not necessarily represent those of their affiliated organizations, or those of the publisher, the editors and the reviewers. Any product that may be evaluated in this article, or claim that may be made by its manufacturer, is not guaranteed or endorsed by the publisher.
