# Supplementary material for: Profile of Basal Cell Carcinoma Mutations and Copy Number Alterations - Focus on Gene-Associated Noncoding Variants
Source: Front Oncol. 2021 Nov 25;11:752579. doi: 10.3389/fonc.2021.752579 (PMC8656283; doi:10.3389/fonc.2021.752579)
Supplement: Supplementary file 1 [file DataSheet_1.pdf]

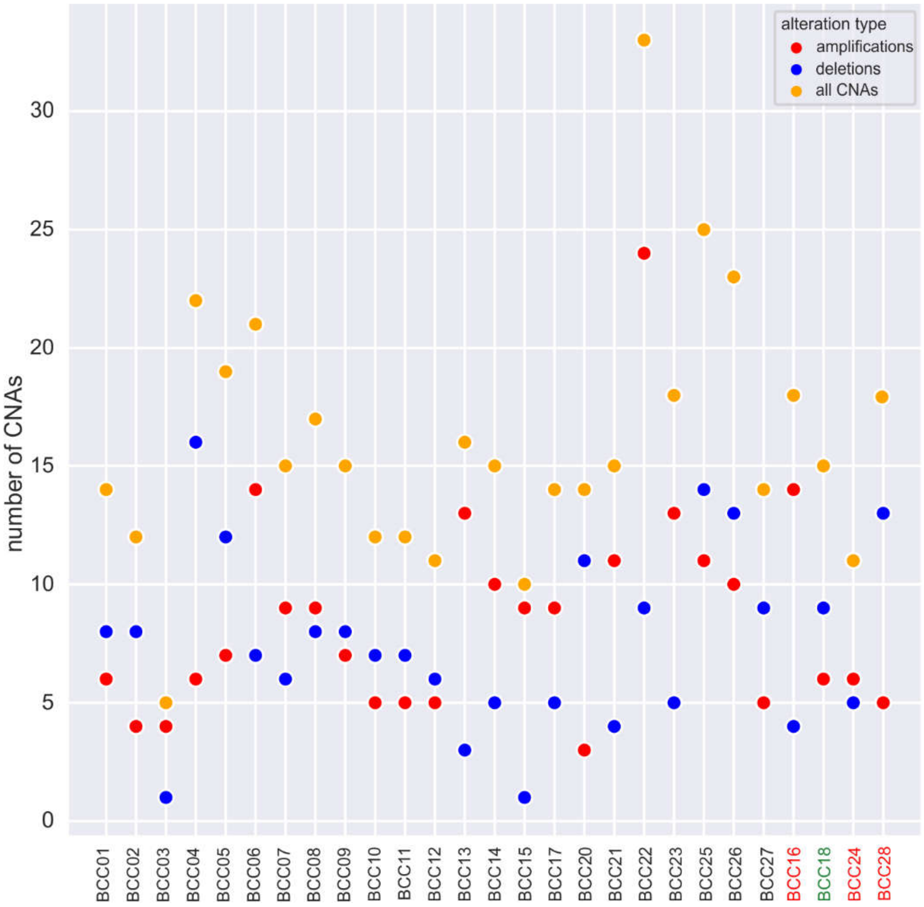

S1 Fig. Concurrence of *KNSTRN* mutations and CNAs. The number of amplifications (red dots), deletions (blue dots), and total CNAs (orange dots) in samples without and with the *KNSTRN* mutations (red fonts - the hotspot mutation; green fonts - other mutation).

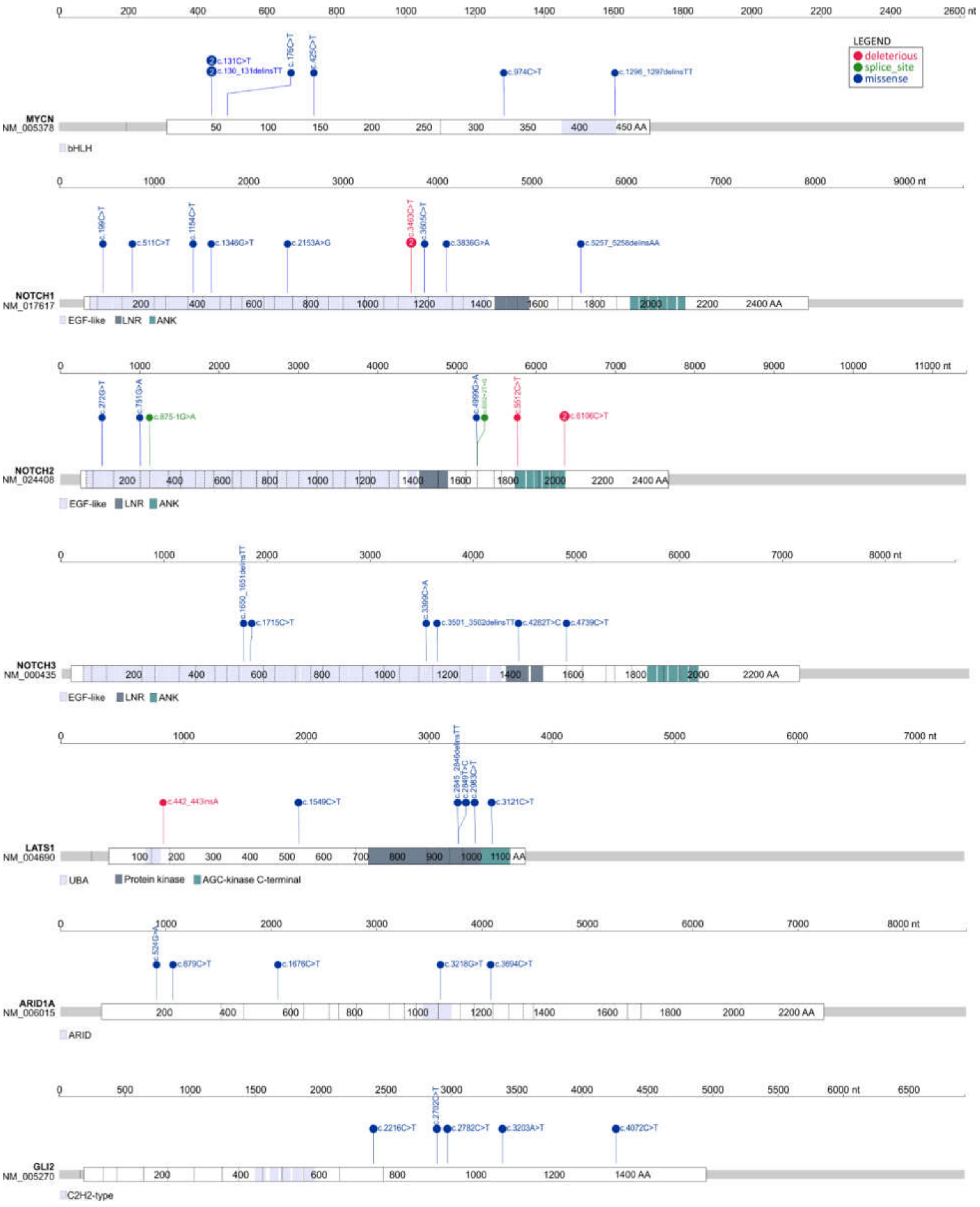

S2 Fig. Distribution of the identified mutations in the selected genes frequently mutated in the coding sequence. Distribution of mutations in the selected genes frequently mutated in coding regions. A)-F) Maps of *MYCN*, *NOTCH1*, *NOTCH2*, *NOTCH3*, *LATS1,* and *ARID1A*, respectively.

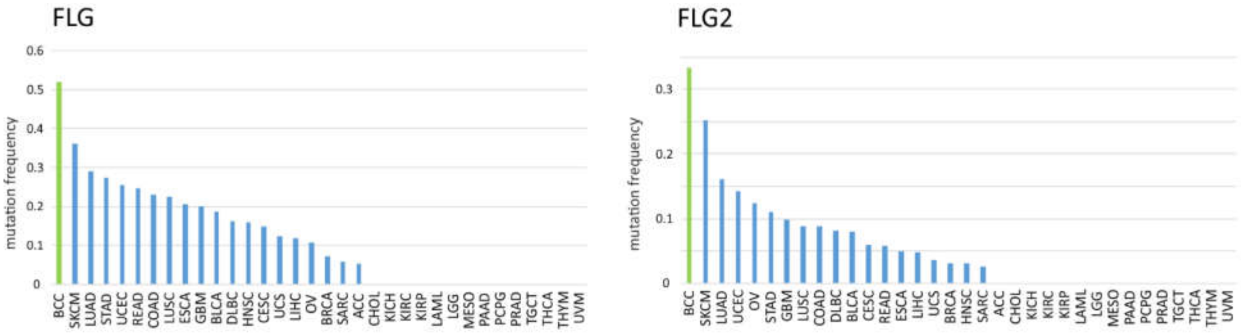

S3 Fig. Frequency of mutations in the *FLG* and *FLG2* genes in BCC and other cancer types. Bar graphs showing the frequency of mutations in A) *FLG*, and B) *FLG2* in BCC (current study, green bar) and TCGA cancer types.

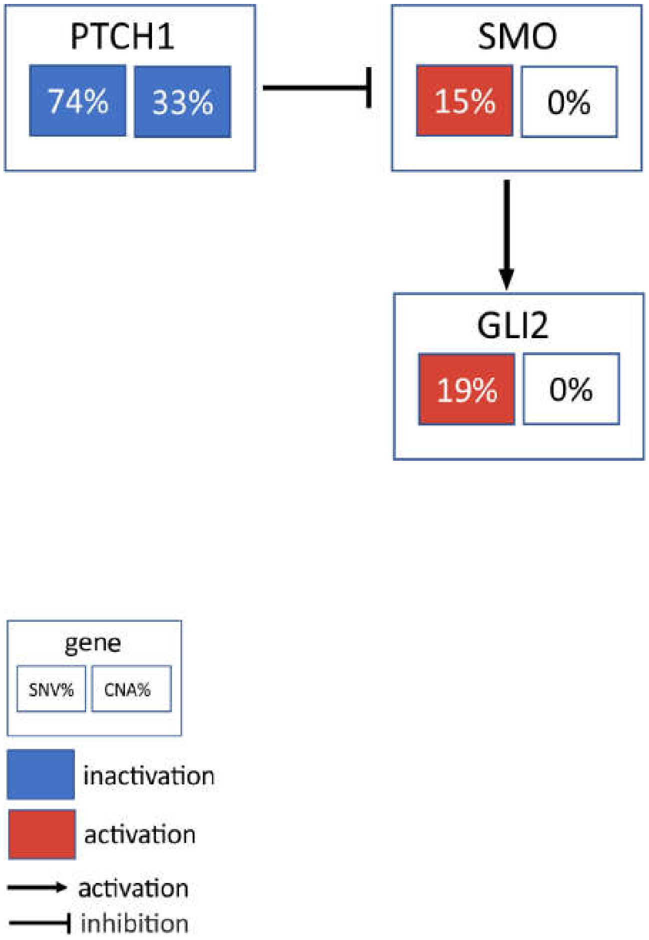

S4 Fig. Frequency of activating (red) and deactivating (blue) mutations in the genes of the hedgehog pathway.
